# Exploration of the binding modes of l-asparaginase complexed with its amino acid substrates by molecular docking, dynamics and simulation

**DOI:** 10.1007/s13205-016-0422-x

**Published:** 2016-04-18

**Authors:** Erva Rajeswara Reddy, Rajulapati Satish Babu, Potla Durthi Chandrasai, Pola Madhuri

**Affiliations:** Department of Biotechnology, National Institute of Technology Warangal, Warangal, 506004 Telangana India

**Keywords:** l-Asparaginase, Erwinaze^®^, Molecular docking, Molecular dynamics and simulation

## Abstract

Acute lymphocytic leukemia (ALL) is an outrageous disease worldwide. l-Asparagine (l-Asn) and l-glutamine (l-Gln) deamination plays crucial role in ALL treatment. Role of Erwinaze^®^ (l-asparaginase from *Erwinia chrysanthemi*) in regulation of l-Asn and l-Gln has been confirmed by the experimental studies. Therapeutic research against ALL remained elusive with the lack of structural information on Erwinaze^®^ enzyme. In this present study, homology model of the Erwinaze^®^ was developed using MODELLER and the same was validated by various quality indexing tools. For the apo state enzyme and ligand bound state complexes molecular dynamics (MD) simulation was performed. The trajectory analysis showed the confirmational changes of structures in the dynamic system. Ligand binding mechanisms were studied using different docking tools to interpret the various ligand-receptor interactions and binding free energies. MD simulation of docked complex with l-Gln ligand substrate showed the defined structural folding with stable conformation over the l-Asn complex in dynamic environment. This research reports give much more information on structural and functional aspects of Erwinaze^®^ with its ligands which may be useful in designing of effective therapeutics for ALL.

**Electronic supplementary material:**

The online version of this article (doi:10.1007/s13205-016-0422-x) contains supplementary material, which is available to authorized users.

## Introduction

Leukemic cell’s inability to synthesize l-Asn on their own has primed l-Asparaginase enzyme as a dynamic factor of chemotherapy in acute lymphoblastic leukemia (ALL) therapy (Ohnuma et al. 1970; Wriston and Yellin 1973). In blood vascular system, l-Asparaginase hydrolyses l-Asn into l-Aspartic acid and ammonia building the leukemic cells barren of essential exogenous l-Asn, leading to protein synthesis inhibition followed by apoptosis (Lubkowski et al. 1994; Neuman and McCoy 1956; Mashburn and Wriston 1964), hence making this potent against ALL.

Nowadays, salable l-Asparaginases are predominantly produced from *E. coli* and *Erwinia chrysanthemi* (Godfrin and Bertrand 2006). Apart from direct production from various bacterial and fungal strains, several attempts were also made for the production of recombinant enzyme by cloning and expression of l-Asparaginase encoding genes from *Erwinia chrysanthemi* (Kotzia and Labrou 2007) and *Erwinia carotovora* (Kotzia and Labrou 2005) and other microbes in *E. coli*. It is found that against the cells of reverted ALL patients, the commercial anti-cancer enzyme has in vitro resistance (Klumper et al. 1995) along with its high glutaminase activity and low substrate specificity, causing pancreatitis, liver dysfunction, coagulation anomalies causing intracranial thrombosis or hemorrhage, neurological seizures and leucopenia (Duval et al. 2002). l-Glutaminase activity of same enzyme leading to deamination of l-Gln to l-Glutamate in blood plasma induces some toxic effects in normal cells (Capizzi and Cheng 1981; Storti and Quaglino 1970). This facilitates the necessity for novel and healthy l-Asparaginases from innocuous microorganisms with elevated substrate affinity, amended stability, low glutaminase activity, adequate half-life and least KM value under physiological conditions to overcome the above said challenges encountered in the recent scenario. Though we have sufficient data on the production, optimization of bioprocess and purification of enzyme etc., no research has been done on the molecular aspects of the enzyme from *Erwinia chrysanthemi*. In the absence of crystal structure it is highly difficult to get the molecular information about the enzyme like its interactions with the substrates, enzyme stability etc. Therefore, the current study is aimed towards the in silico modeling of l-Asparaginase structure from *Erwinia chrysanthemi*, molecular interactions with the substrates through docking, and testing the stability of the enzyme and docked complexes under physiological conditions by molecular dynamic simulations methods.

## Materials and methods

### Preparation of receptor and ligands

Ligand molecules l-Asn (C_4_H_8_N_2_O_3_) and l-Gln (C_5_H_10_N_2_O_3_) whose molecular masses are 132.12 g/mol and 146.14 g/mol were retrieved from Zinc database with ID numbers 1532556 and 1532526, respectively. Then they were subjected to energy minimization using the MMFF (Merck Molecular Force Field) (Halgren 1996) of VLifeMDS v 4.3 that works based on MM3 force fields until reaching global minima.

Homology modeling approach was used to investigate the tertiary structure of the enzyme. Hypothetical configuration of enzyme was obtained by MODELLER v 9.13 tool using amino acid sequence provided by Drugbank (http://www.drugbank.ca/drugs/DB08886). Further the modeled enzyme was validated using Ramachandran plot analysis by Rampage (Lovell et al. 2003), followed by determination of QMEAN 6 score (Benkert et al. 2008), DFire energy value (Zhou and Zhou 2002) using SWISS-MODEL server and ERRAT 2.0 (Colovos and Yeates 1993) tools to verify the stereochemical quality of the model by analyzing the phi (ø) and psi (ψ) torsion angles, estimation of local quality of the modeled enzyme, assessment of non-bonded atomic interactions and for appraising the growth of crystallographic model construction and refinement, respectively.

### Molecular docking

iGEMDOCK v 2.1 is a graphical atmosphere for identifying the pharmacological interactions and virtual screening that are beneficial for pinpointing the lead compounds and understanding the mechanism of ligand binding against a therapeutic target. iGEMDOCK, a flexible docking tool can be used for the docking, virtual screening and post-screening analysis. The post analysis tools works by using K means and hierarchal clustering methods (Hsu et al. 2011).

Interactive interface was provided initially for preparing target protein’s binding site and compound library screening in GEMDOCK. The complete modeled structure of Erwinaze^®^ was uploaded in the “Prepare Binding Site” and the “By current file” choice was kept because the uncut surface will be checked for binding, instead of specifying a single cavity. Then the in-house docking tool GEMDOCK docks the compounds from library into receptor binding site. Protein-compound interaction profiles were generated and analyzed by post analysis tools. iGEMDOCK finally ranks and visualizes the compound based on energy based scoring function and pharmacological interactions (Kaladhar 2012). During docking process, GA parameters were set as population size of 300, generations of 80 and solutions number as 10. Stable docking (slow) was performed with the docking scoring function as GEMDOCK scoring function and 1.00 was set to ligand hydrophobic and electrostatic preferences. Low energy profile indicates the stable system and it represents the likely binding interaction.

To verify the results obtained by iGEMDOCK, again molecular docking was performed by Patch Dock server by uploading the structures to web server (Schneidman-Duhovny et al. 2005) that works based on shape complementarity principles and again the outcomes were refined with FireDock server (Andrusier et al. 2007; Mashiach et al. 2008) that reshuffles the interface side chains and amends the molecule’s relative orientation. Analysis of ligand binding interactions and docking viability was established on Fire Dock scores and visualizations with Pymol. Docking parameters were set as 30˚ as rotation angle, 30 as number of placements and 10 as ligand wise results. Intel Core i7 7470 CPU @ 3.40 GHz of DELL origin, with 8 GB DRR RAM under Windows 8.1 OS was used to perform all the docking runs.

### Molecular dynamics and simulation

MD simulations were executed for the homology modeled Erwinaze^®^ enzyme, Erwinaze^®^-l-Asn (complex 1) and Erwinaze^®^-l-Gln (complex 2) docked complexes developed from molecular docking to ratify the stability for anti-cancer enzyme in apo state and bound state with the substrates in dynamic system. Generating the l-Asn and l-Gln topologies using PRODRG server was the early step in MD simulations (SchuÈttelkopf and Van Aalten 2004). After defining ligand topologies, MD simulation for modeled enzyme and docked complex were carried using GROMACS 4.6.5 program package under Ubuntu 14.04 operating system. Steepest algorithm using the GROMOS 96 43a1 (van Gunsteren et al. 1996; Scott et al. 1999) was used for energy minimization, dismissing when the maximum force was found lesser than 1000 kJ mol^−1^ nm^−1^. The modeled enzyme and the docked complexes were solvated in the system of a cubic box with a size of 1.0 nm and at least 2.0 nm between any two periodic images of a protein to provide an aqueous environment. With the addition of one chlorine ion the system was neutralized and periodic boundary conditions were applied in all directions. The cubic interpolation order in particle mesh Ewald (PME) simulation method was 4 and the grid spacing for FFT (Fourier spacing) was 0.16. In the neighbor searching method, the short range neighbor list cutoff of 1 Å was taken commonly for electrostatic interactions and Vander Walls interactions. The LINCS (Hess et al. 1997) and SETTLE algorithms (Miyamoto and Kollman 1992) were applied to constrain all bond lengths and geometry of water molecules, respectively. For 100 ps duration, the two equilibration phases NVT ensemble with a constant temperature of 300 K, coupling constant of 0.1 ps and NPT ensemble with constant pressure of 1 bar, coupling constant of 2 ps were applied for all the molecules. Modified Berendsen thermostat coupling scheme algorithm was employed for both ensembles of equilibration. Once the system equilibration with constant temperature and pressure was done, to carry out the structural and energy analyses30 ns production MD run was performed. Run trajectories were obtained and with GROMACS utilities analysis was carried out. Using g_energy, g_rms, g_rmsf and g_gyrate quality assurance of all the molecules was performed. g_hbond was used to cross check the docking results in terms of hydrogen bonding pattern between the receptor and ligand substrates. Using Xmgrace tool the entire trajectory results were analyzed (Turner 2005).

## Results and discussion

### Molecular modeling of l-asparaginase from *Erwinia chrysanthemi*

For predicting protein three-dimensional structure based on user provided alignment of a sequence to be modeled with known related structures, homology or comparative modeling tool MODELLER can be used. BLAST search was performed to identify the most similar sequences whose crystal structures were experimentally determined. Search results suggested 1HFJ_A, 1O7J_A, 1ZCF_A and 2JK0_A as potential templates of bacterial origin having 99, 98, 79 and 79 % of sequence identity and SuperPose (http://wishart.biology.ualberta.ca/superpose/) gave the backbone RMSD values of 0.31, 0.37, 0.83 and 0.43 nm, respectively, from the query. Calculation of a model containing all non-hydrogen atoms was automatically done by MODELLER using all the four templates to cover the complete query sequence. Best homology model was chosen based on the low discrete optimized protein energy (DOPE) score and RMSD values amongst a total of ten models predicted by MODELLER (Šali and Blundell 1993; Fiser et al. 2000).

**Fig. 1:**
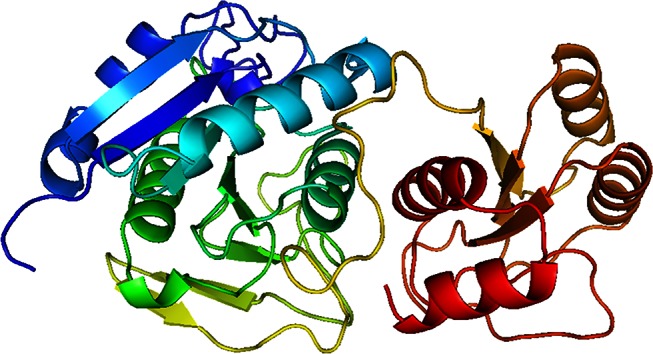
Structure of Erwinaze^®^ predicted by MODELLER

The hypothetical model of Erwinaze^®^ enzyme (Fig. 1) was validated using Rampage geometric evaluations to get Ramachandran plot. The plot has 97.2, 2.8 and 0.0 %of residues in favored, allowed and disallowed regions, respectively, and this strongly supports the geometric fitness of the modeled enzyme. Qmean6 score of 0.671, DFire energy of −455.15 and 84.326 % overall quality factors from ERRAT 2.0 indicates the good resolution structure. Secondary structure of the protein was also analyzed using PredictProtein tool (http://www.predictprotein.org). Protein was rich in loop region with a percentage of 46.01 residues and the remaining 29.14 and 24.85 % ones as helix and strands with two N-glycosylation sites in it. Along with these it also has eight sites for protein kinase C phosphorylation, three sites for Casein kinase II phosphorylation and one Tyrosine kinase phosphorylation. The final structure was further used to study the molecular interactions with ligand molecule.

### Docking with l-Asn and l-Gln

Two ligand substrates namely l-Asn and l-Gln were docked into the catalytic site of Erwinaze^®^. Dock runs of ligands on the enzyme were done using iGEMDOCK v 2.1 and PatchDock server. When docking runs were carried out by iGEMDOCK, Erwinaze^®^ has shown very high binding affinity with l-Asn and l-Gln (−52.6373 kcal/mol and −57.2457 kcal/mol, respectively). Results obtained by iGEMDOCK were again validated by PatchDock webserver followed by refinement of obtained results with FireDock server.

Outcomes strongly supported the previous results with a very good binding efficiency between receptor and ligands with the calculated global energy values of −20.41 kcal/mol and −25.32 kcal/mol for l-Asn and l-Gln, respectively, by FireDock server. l-Asn made inter molecular hydrogen bonds with THR-15, THR-26, THR-27 and MET-121 of Erwinaze^®^ and on the other side L-Gln generated bonds with THR-165, THR-167 and ASN-180 of same enzyme (Table 1). In fact, the enzyme has more affinity towards l-Asn; as it has formed the four inter molecular hydrogen bonds with the ligand atoms with the distances of 2.6, 2.5, 3.4 and 2.7 Å which are lesser compared to the bond lengths resulted with l-Gln.

**Table 1:** Molecular Interactions between Erwinaze^®^ and ligand substrates

S.No.	Ligand	Residue and atom	Ligand atom	Bond length (Å)
1	l-Asn	THR15 & OG_1_	O_3_	2.6
2		THR26 & O	N_1_	2.5
3		THR27 & OG_1_	N_1_	3.4
4		MET121 & O	N_2_	2.7
5	l-Gln	TYR165 & O	N_2_	2.6
6		THR167 & OG_1_	N_2_	3.5
7		THR167 & N	O_2_	2.9
8		ASN180 & N	O_3_	3.3

**Fig. 2:**
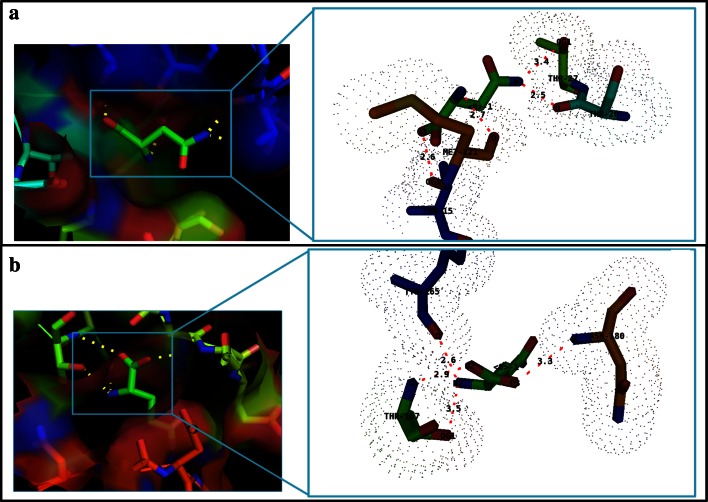
Molecular docking results of Erwinaze^®^ with ligand substrates **a** binding with l-Asn, **b** binding with l-Gln

Summary of the binding energies by all the docking methods were described in Table 2. The mode of binding and cavity of ligands with receptor was visualized in PyMol molecular graphics viewer (Fig. 2). Cross checking of the modeled enzyme binding sites in docking was done with active site prediction tool (Ngan et al. 2012; Brenke et al. 2009). Pooled results exhibited matching of most of the residues, and thus, the binding sites used in docking approach were well defined (Supplementary material). As the exertion of biological activity of any drug is based on the binding between protein and ligand, present investigation results reveal that the nearly equal affinity of Erwinaze^®^ towards both the ligands strongly supports Asparaginase and Glutaminase activities of the enzyme (Keating et al. 1993; Derst et al. 2000). Numerous adverse effects of l-Asparaginase usage have also been stated, distant from severe immunogenic reactions (Kwon et al. 2009).

**Table 2:** Molecular Docking results of Erwinaze^®^ with ligand substrates

Docking tool	Ligand	
	l-Asn (kcal/mol)	l-Gln (kcal/mol)
iGEMDOCK	−52.6373	−57.2457
PatchDock and FireDock	−20.41	−25.321

### MD simulations: protein in water

**Fig. 3:**
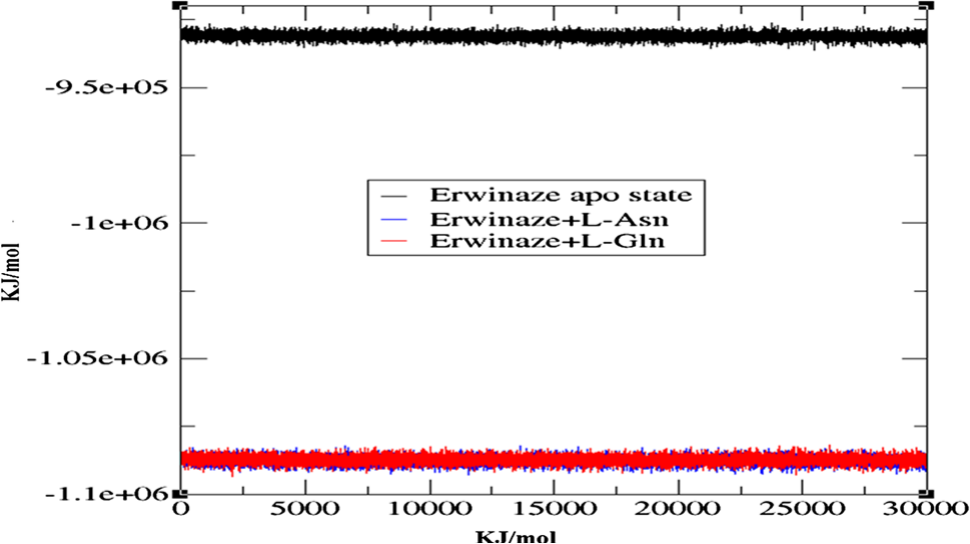
Total energy of Erwinaze^®^ apo enzyme after molecular dynamics and simulations

**Fig. 4:**
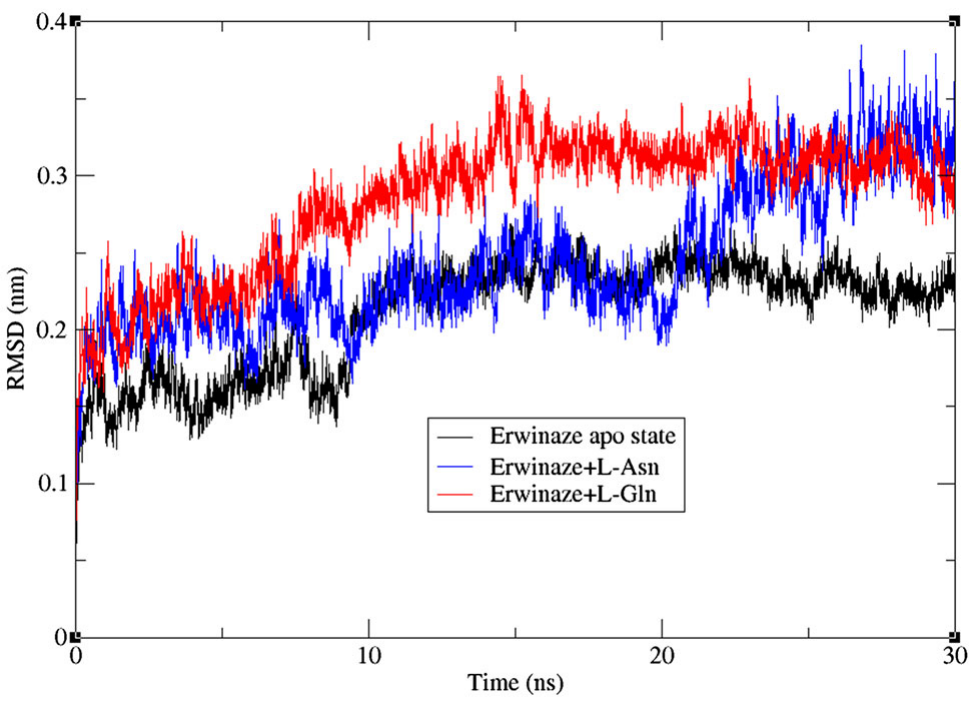
RMSD of backbone atoms for apo state enzyme, Erwinaze^®^ + l-Asn and Erwinaze^®^ + l-Gln complexes

To check the structural behavior of modeled enzyme and docked complexes in the dynamic system MD simulations were performed and the trajectory analysis was done using GROMACS utilities. The total energy found from g_energy results for Erwinaze^®^ modeled enzyme was found to be −9,30,919 kJ/mol demonstrating that the model was energetically stable (Fig. 3). The structural convergence includes terms like root mean square deviation (RMSD), root mean square fluctuation (RMSF), radius of gyration (Rg) and hydrogen bond (H-bond) analysis. In the g_rms based results with backbone atoms (Fig. 4), apo state enzyme is stable right from the launch of MD run with a drift at 10 ns followed by a stable confirmation till the finale of entire run.

In case of docked complexes, though the complex 1 is unwavering till 20 ns it exhibited an increasing path later and extended the RMSD value to virtually 0.4 nm. RMSD of complex 1 with l-Asn till 10 ns specified the ligand binding in apo state and after 10 a significant conformational change with deviation was observed which might be the plausible cause to obtain l-Asn bound state. Its high RMSD value shows the instability of the complex which is undesirous. On the other hand, complex 2 has shown a perpetual RMSD value around 0.3 nm showing its high stability in the 300°K atmosphere supporting the experimental results of neurotoxicity (Capizzi and Cheng 1981; Storti and Quaglino 1970) of l-Glutaminase nature of enzyme. This leaves the plasma pool l-Asn free for the uptake by cancerous cells on one hand because of unstable nature of complex 1, and causing the toxicity in normal cells due to its stable Glutaminase activity. Both the complexes are displaying their undesirable results against the actual desired l-Asparaginase role of enzyme in cancer therapy.

**Fig. 5:**
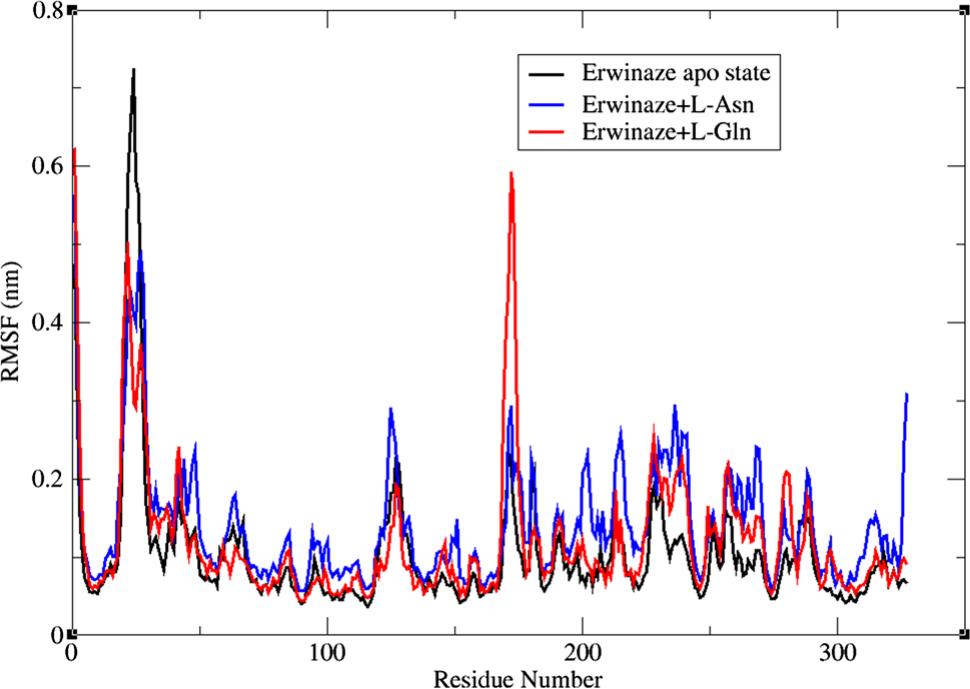
RMSF of Cα atoms for apo state enzyme, Erwinaze^®^ + l-Asn and Erwinaze^®^ + l-Gln complexes

In the analysis of g_rmsf results, many oscillations in the residues with Cα atoms were observed in case of first complex with a fall and amendment in initial peak due to the binding of l-Asn in that region. This substrate binding also influenced the entire enzyme with several numbers of residual fluctuations with a near RMSF value of 0.3 nm (Fig. 5). The same plot also disclosed the presence of a second high peak in the vicinity of bounded residues establishing H-bonds with ligand leading to a very high RMSF value of around 0.6 nm in complex 2, leaving most of the other residues stable. The comparison of RMSF outcomes exhibited minor variations in ligand binding sites (Table 3) and their effect on complex formation.

**Table 3:** Comparison of RMSF values from MD simulations

S. no.	Residue	RMSF (nm)		
		Erwinaze^®^ in apo state	Complex 1	Complex 2
1	TYR15	0.0905	0.1108	0.0832
2	THR 26	0.5626	0.4778	0.3424
3	THR27	0.4196	0.4855	0.3730
4	MET121	0.1318	0.1355	0.0938
5	TYR165	0.0647	0.0736	0.0677
6	THR167	0.0616	0.0839	0.0722
7	ASN180	0.1866	0.2255	0.1255

**Fig. 6:**
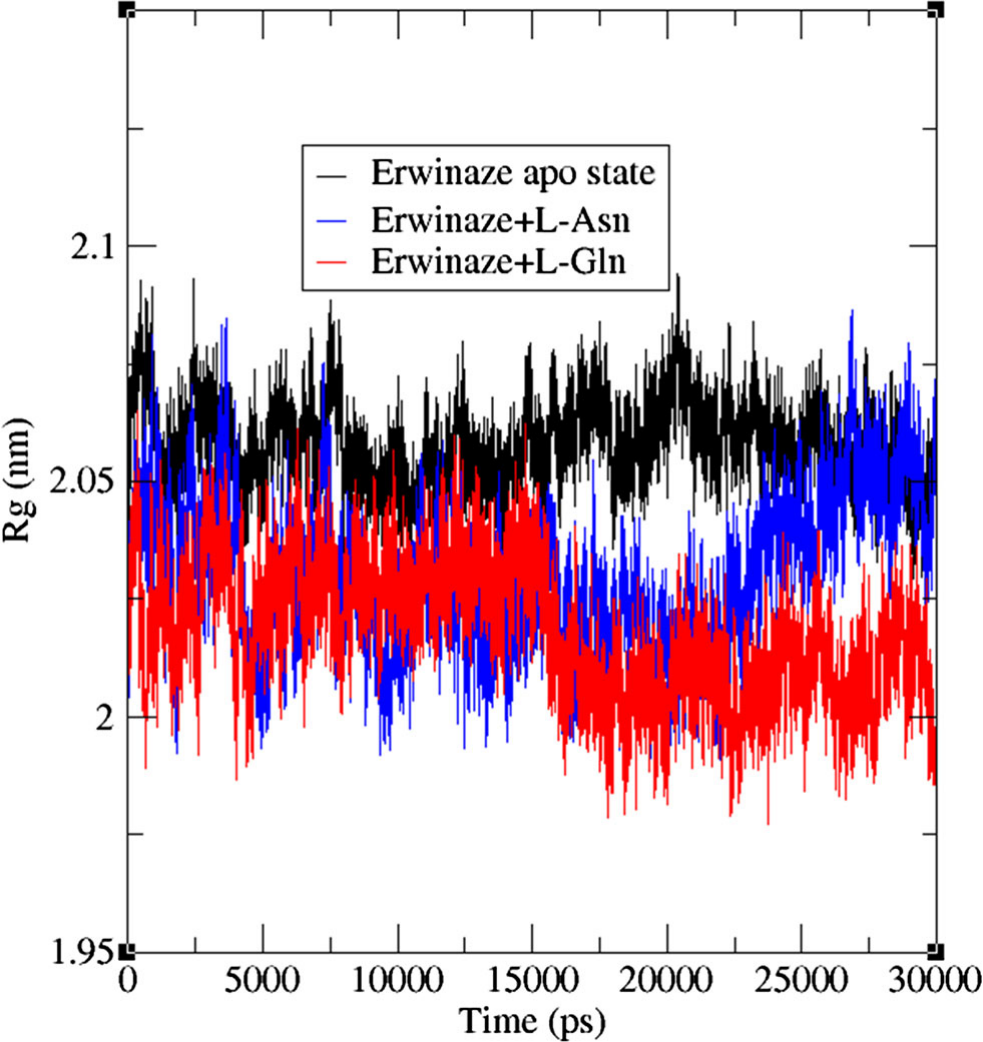
Rg plots for apo state enzyme, Erwinaze^®^ + l-Asn and Erwinaze^®^ + l-Gln complexes

Radius of gyration (Rg) was done to analyze the structural compactness of enzyme and was calculated using g_gyrate tool of GROMACS. Though the modeled enzyme in its free state has some structural compactness, bounded complexes were shown their fluctuating nature from Rg plots. Complex 1 has displayed the drifts during entire MD run in the array of 2.0 and 2.08 nm. In the plot of complex 2, it revealed its compactness with a little drift at 15 ns time point with the Rg values oscillating between 2.0 and 2.06 nm (Fig. 6).

**Fig. 7:**
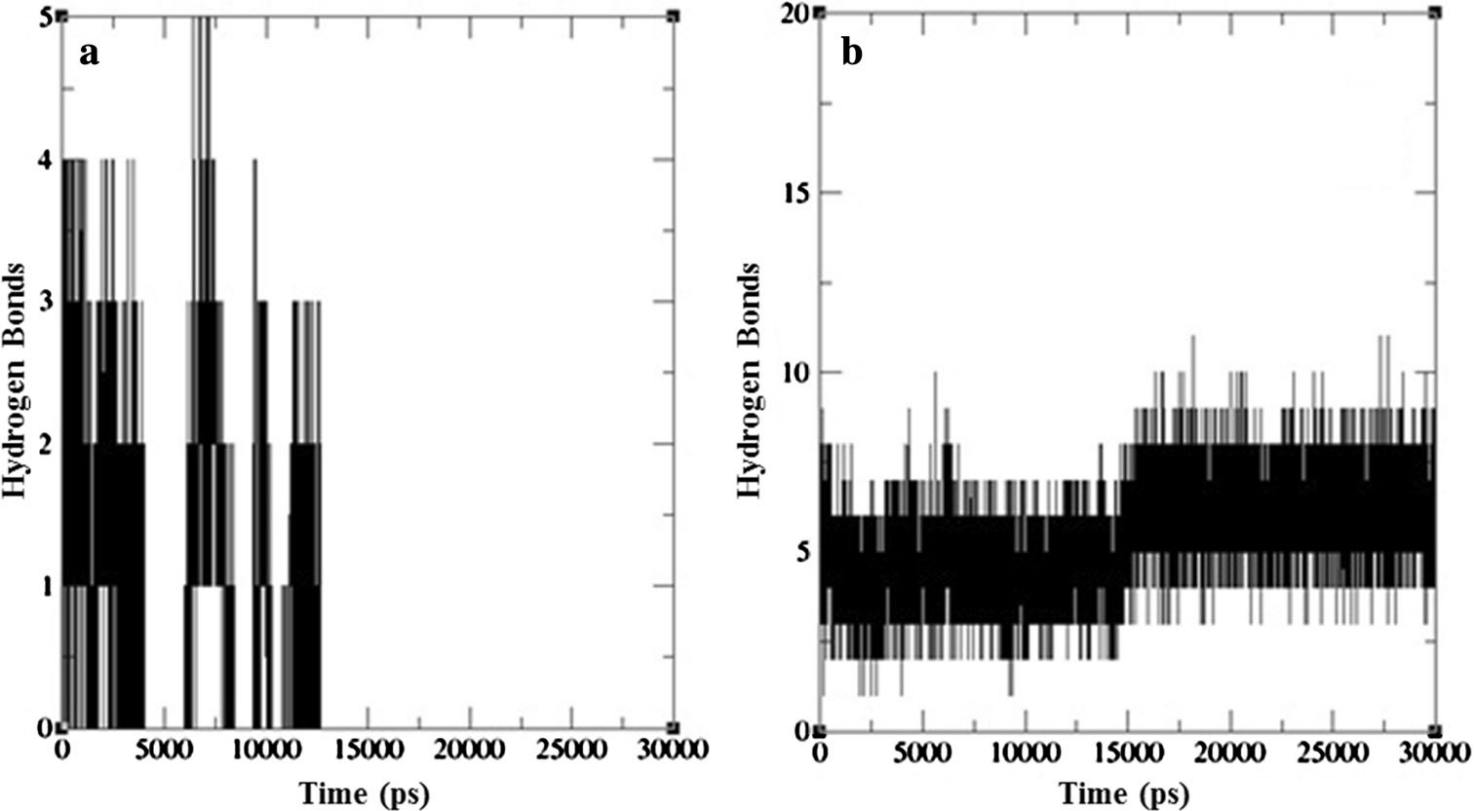
Inter-hydrogen bonding for docked complexes **a** Erwinaze^®^ + l-Asn, **b** Erwinaze^®^ + l-Gln

To authenticate the hydrogen bonding pattern docking results, H-bond analysis was also executed along with the intact MD trajectories (Fig. 7). Surprisingly, the desired stable H-bonding pattern for complex 1 described missing linkage of hydrogen bonds between receptor and ligand. During 7.5 ns point the H-bonds were increased to 5, but after 4.03 ns h-bonds were missed and again formed from 6 ns. The same was again repeated at around 9 ns and 11 ns time points. Finally, after 12.6 ns there were no h-bonds, showing the disappearance of H-bond linkage between enzyme and substrate (Fig. 7a). The loss of intermolecular hydrogen bands may induce a spatial conformational change in the tertiary structure of the enzyme i.e., unstable structure of docked complex. This becomes a major shortcoming for the enzyme in therapeutic perspective, supporting possibility of its lower effect on cancer cells. On the other hand, it has shown the constant H-bond pattern with l-Gln throughout MD run with an average of 7 H-bonds throughout entire trajectory (Fig. 7b). H-bond pattern of both complexes did not draw a parallel with the docking result and this gives scope for the further investigation for efficient and firm drug in ALL therapy. Entire computational analysis describes the necessity of further intensive investigation on other sources of l-Asparaginase enzyme which is more effective on cancerous cells with fewer side effects.

## Conclusion

l-Asparaginase is one of the most attractive enzymes in cancer research. Investigational studies on Erwinaze^®^ sustained it as one of the therapeutic agent for ALL. In the present computational study, hypothetical model of Erwinaze^®^ enzyme was developed and MD simulations for the same in apo state confirmed its stability. Ligand binding studies on Erwinaze^®^ with l-Asn and l-Gln using molecular docking explained the binding mechanism of docked complexes. Key residues in binding were identified as Thr 15, Thr 26, Thr 27and Met 121 in l-Asn binding followed by Tyr 165, Thr 167 and Asn 180 in l-Gln binding based on the docking results. MD simulations of complex 1 for 30 ns run confirmed the instability and unlikeliness of enzyme towards l-Asn and the kinship with l-Gln in the dynamic system with stability. Quite a few conformational changes in enzyme were observed with Erwinaze^®^ structure due to the l-Asn ligand binding compared to l-Gln binding. This in silico studies favor added extensive structural research on l-Asparaginase towards scheming of potential inhibitors that aim the l-Asparaginase for effective treatment of ALL.

## Electronic supplementary material

Below is the link to the electronic supplementary material.

Supplementary material 1 (PDF 368 kb)

## References

[CR1] Andrusier N, Nussinov R, Wolfson HJ (2007). FireDock: fast interaction refinement in molecular docking. Proteins Struct Funct Bioinform.

[CR2] Benkert P, Tosatto SC, Schomburg D (2008). QMEAN: a comprehensive scoring function for model quality assessment. Proteins Struct Funct Bioinform.

[CR3] Brenke R (2009). Fragment-based identification of druggable ‘hot spots’ of proteins using Fourier domain correlation techniques. Bioinformatics.

[CR4] Capizzi R, Cheng Y, Holcenberg JS, Roberts J (1981). Therapy of neoplasia with asparaginase. Enzymes as Drugs.

[CR5] Colovos C, Yeates TO (1993). Verification of protein structures: patterns of non-bonded atomic interactions. Protein Sci.

[CR6] Derst C, Henseling J, Röhm KH (2000). Engineering the substrate specificity of Escherichia coli asparaginase II. Selective reduction of glutaminase activity by amino acid replacements at position 248. Protein Sci.

[CR7] Duval M (2002). Comparison of *Escherichia coli*–asparaginase withErwinia-asparaginase in the treatment of childhood lymphoid malignancies: results of a randomized European Organisation for Research and Treatment of Cancer—Children’s Leukemia Group phase 3 trial. Blood.

[CR8] Fiser A, Do RKG, Šali A (2000). Modeling of loops in protein structures. Protein Sci.

[CR9] Godfrin Y, Bertrand Y (2006). l-asparaginase introduced into erythrocytes for the treatment of leukaemia (ALL). BioMedES.

[CR10] Halgren TA (1996). Merck molecular force field. I. Basis, form, scope, parameterization, and performance of MMFF94. J Comput Chem.

[CR11] Hess B, Bekker H, Berendsen HJ, Fraaije JG (1997). LINCS: a linear constraint solver for molecular simulations. J Comput Chem.

[CR12] Hsu K-C, Chen Y-F, Lin S-R, Yang J-M (2011). iGEMDOCK: a graphical environment of enhancing GEMDOCK using pharmacological interactions and post-screening analysis. BMC Bioinform.

[CR13] Kaladhar D (2012). An in vitro callus induction and isolation, identification, virtual screening and docking of drug from convolvulus alsinoides linn against aging diseases. Int J LifeSc Bt Pharm Res.

[CR14] Keating MJ, Holmes R, Lerner S, Ho DH (1993). l-asparaginase and PEG asparaginase-past, present, and future. Leuk Lymphoma.

[CR15] Klumper E (1995). In vitro cellular drug resistance in children with relapsed/refractory acute lymphoblastic leukemia. Blood.

[CR16] Kotzia GA, Labrou NE (2005). Cloning, expression and characterisation of Erwinia carotovoral-asparaginase. J Biotechnol.

[CR17] Kotzia GA, Labrou NE (2007). l-Asparaginase from Erwinia chrysanthemi 3937: cloning, expression and characterization. J Biotechnol.

[CR18] Kwon YM (2009). l-Asparaginase encapsulated intact erythrocytes for treatment of acute lymphoblastic leukemia (ALL). J Controll Releas.

[CR19] Lovell SC (2003). Structure validation by Cα geometry: ϕ, ψ and Cβ deviation. Proteins Struct Funct Bioinform.

[CR20] Lubkowski J, Wlodawer A, Housset D, Weber IT, Ammon H, Murphy K, Swain A (1994). Refined crystal structure of Acinetobacter glutaminasificans glutaminase–asparaginase. Acta Crystallogr D Biol Crystallogr.

[CR21] Mashburn LT, Wriston JC (1964). Tumor inhibitory effect of l-asparaginase from *Escherichia coli*. Arch Biochem Biophys.

[CR22] Mashiach E, Schneidman-Duhovny D, Andrusier N, Nussinov R, Wolfson HJ (2008). FireDock: a web server for fast interaction refinement in molecular docking. Nucl Acids Res.

[CR23] Miyamoto S, Kollman PA (1992). SETTLE: an analytical version of the SHAKE and RATTLE algorithm for rigid water models. J Comput Chem.

[CR24] Neuman RE, McCoy TA (1956). Dual requirement of Walker carcinosarcoma 256 in vitro for asparagine and glutamine. Science.

[CR25] Ngan C-H, Hall DR, Zerbe B, Grove LE, Kozakov D, Vajda S (2012). FTSite: high accuracy detection of ligand binding sites on unbound protein structures. Bioinformatics.

[CR26] Ohnuma T, Holland JF, Freeman A, Sinks LF (1970). Biochemical and pharmacological studies with asparaginase in man. Cancer Res.

[CR27] Šali A, Blundell TL (1993). Comparative protein modelling by satisfaction of spatial restraints. J Mol Biol.

[CR28] Schneidman-Duhovny D, Inbar Y, Nussinov R, Wolfson H (2005). Nucl Acids Res.

[CR29] SchuÈttelkopf AW, Van Aalten DM (2004). PRODRG: a tool for high-throughput crystallography of protein–ligand complexes. Acta Crystallogr D Biol Crystallogr.

[CR30] Scott WR (1999). The GROMOS biomolecular simulation program package. J Phys Chem A.

[CR31] Storti E, Quaglino D (1970). Dysmetabolic and neurological complications in leukemic patients treated with l-asparaginase. Experimental and clinical effects of L-asparaginase.

[CR32] Turner P (2005) XMGRACE, Version 5.1. 19 center for coastal and land-margin research. Oregon Graduate Institute of Science and Technology, Beaverton

[CR33] van Gunsteren WF et al. (1996) Biomolecular simulation: the {GROMOS96} manual and user guide. ETH Zürich, Zürich

[CR34] Wriston J, Yellin T (1973). l-asparaginase: a review. Adv Enzymol Relat Areas Mol Biol.

[CR35] Zhou H, Zhou Y (2002). Distance-scaled, finite ideal-gas reference state improves structure-derived potentials of mean force for structure selection and stability prediction. Protein Sci.

