# Supplementary material for: Exploration of the binding modes of l-asparaginase complexed with its amino acid substrates by molecular docking, dynamics and simulation
Source: 3 Biotech. 2016 Apr 18;6(1):105. doi: 10.1007/s13205-016-0422-x (PMC4835421; doi:10.1007/s13205-016-0422-x)

FT Site Prediction for ERWINAZE by FT Site

Site 1 Residues	Site 2 Residues	Site 3 Residues
ASP X 96	ARG X 164	ASP X 96
GLU X 99	TYR X 165	THR X 97
GLU X 100	ILE X 166	GLU X 99
THR X 167	THR X 167	GLU X 100
LYS X 168	LYS X 178	ALA X 301
THR X 169	ALA X 179	
ASN X 170	ASN X 180	
ALA X 171	GLU X 181	
THR X 176	GLU X 182	
ASN X 180	GLY X 280	
ILE X 222	ASN X 281	
LEU X 223	GLY X 282	
TYR X 224	SER X 297	
ALA X 248	LEU X 298	
THR X 277	ASN X 299	
ARG X 278	HIS X 302	
THR X 279		
GLY X 280		
ASN X 281		
ASN X 299		
PRO X 300		
ALA X 301		
ARG X 304		

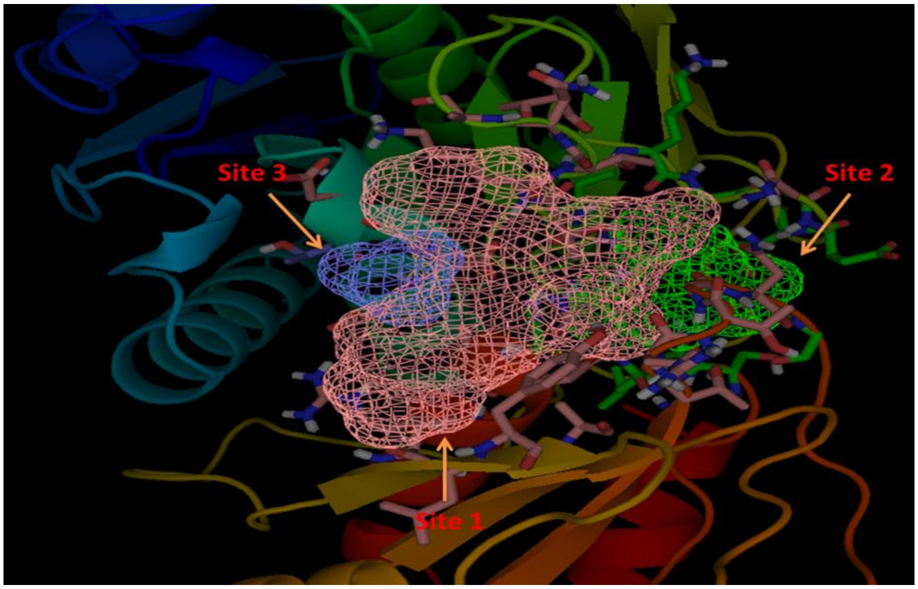

Supplement: Supplementary file 1 — Supplementary material 1 (PDF 368 kb) [file 13205_2016_422_MOESM1_ESM.pdf]
